# Dissecting geographic variation in population synchrony using the common vole in central Europe as a test bed

**DOI:** 10.1002/ece3.1863

**Published:** 2015-12-17

**Authors:** Ana R. Gouveia, Ottar N. Bjørnstad, Emil Tkadlec

**Affiliations:** ^1^ Department of Ecology and Environmental SciencesFaculty of SciencePalacky University OlomoucŠlechtitelů 2777146OlomoucCzech Republic; ^2^ Departement of Entomology and the Centre for Infectious Disease Dynamicsthe Pennsylvania State UniversityState CollegePennsylvania16802; ^3^ Institute of Vertebrate BiologyAcademy of Sciences of the Czech RepublicKvětná 8603 65BrnoCzech Republic

**Keywords:** Altitudinal gradient, LISA, *Microtus arvalis*, partial nonparametric correlation function, spatiotemporal dynamics

## Abstract

Spatial synchrony of population fluctuations is ubiquitous in nature. Theoretical models suggest that correlated environmental stochasticity, dispersal, and trophic interactions are important promoters of synchrony in nature to leave characteristic signatures of distance‐dependent decays in synchrony. Recent refinements of this theory have clarified how distance‐decay curves may steepen if local dynamics are governed by different density‐dependent feedbacks and how synchrony should vary regionally if the importance and correlation of environmental stochasticity is location‐specific. We analysed spatiotemporal data for the common vole, *Microtus arvalis* from 49 districts in the Czech Republic to examine the pattern of population synchrony between 2000 and 2014. By extending the nonparametric covariation function, we develop a quantitative method that allows a dissection of the effects of distance and additional variables such as altitude on synchrony. To examine the pattern of local synchrony, we apply the noncentered local‐indicators of spatial association (ncLISA) which highlights areas with different degrees of synchrony than expected by the region‐wide average. Additionally, in order to understand the obtained pattern of local spatial correlations, we have regressed LISA results against the proportion of forest in each district. The common vole abundances fluctuated strongly and exhibited synchronous dynamics with the typical tendency for a decline of synchrony with increasing distance but, not with altitude. The correlation between the neighbor districts decreases as the proportion of forest increases. Forested areas are suboptimum habitats and are strongly avoided by common voles. The investigation of spatiotemporal dynamics in animal populations is a key issue in ecology. Although the majority of studies are focused on testing hypotheses about which mechanisms are involved in shaping this dynamics it is crucial to understand the sources of variation involved in order to understand the underlying processes.

## Introduction

Correlation in population dynamics over large geographical areas also known as spatial synchrony has been extensively studied in a variety of taxa including insects (Pollard 1991; Hanski and Woiwod 1993; Sutcliffe et al. 1996, 1997; Bjørnstad et al. 2002; Peltonen et al. 2002, Tenow et al. 2007, Haynes et al. 2013), birds (Ranta et al. 1995b; Lindström et al. 1996; Jones et al. 2008), and mammals (Bondrup‐Nielsen and Ims 1988; Hansson 1990; Mackin‐Rogalska and Nabagło 1990; Bjørnstad et al. 1999a; 2008, Krebs et al. 2002; Grøtan et al. 2005). Generally, there are three main recognized and widely studied promotors of synchrony in animal populations: dispersal, trophic interactions, and correlated environmental stochasticity. Dispersal of individuals from the population can act as synchronising agents (Liebhold et al. 2004, 2006), usually at local scales. In contrast highly mobile predators and climate effects are thought to act at larger regional scales. Different trophic interactions have also been reported as playing a role in synchronizing populations such as predator–prey interactions (Ims and Steen 1990; Heikkilä et al. 1994; de Roos et al. 1998; Gurney et al. 1998; Bjørnstad et al. 1999b; Ims and Andreassen 2000; Korpimäki et al. 2005). Both Heikkilä et al. (1994) and Korpimäki et al. (2005) have shown that the microtine rodent low phase may be synchronized by predators (mustelids and birds). A vast amount of research suggests the effect of environmental stochasticity, the Moran's theorem, as the main or at least most studied mechanism. This theorem states that spatial synchrony of fluctuating local populations with a shared density dependent structure is the result of the influence of correlated environmental forces affecting local demographics (Moran 1953a,b; Ranta et al. 1995a; Hudson and Cattadori 1999; Ripa 2000). Regardless of the mechanism, most studies conclude that spatial synchrony should typically decrease with geographic distance (Bjørnstad et al. 1999b; Koenig 1999; Lundberg et al. 2000; Liebhold et al. 2004; Carslake et al. 2011), though U‐shaped patterns have been described for some system with complex spatiotemporal dynamics (Ranta et al. 1997b; Bjørnstad and Bascompte 2001).

The study of synchrony in animal populations has employed a combination of theoretical and experimental approaches, using a variety of statistical tools to bridge between the two. Typically the statistical characterization of spatial synchrony between two or more populations is done by first generating a matrix of Pearson or Spearman product‐moment correlation coefficient among time series of abundances or growth rates (Bjørnstad et al. 1999b). This is then followed by either a parametric or nonparametric regression, with nonparametric approaches of correlation coefficients against distance being most commonly used nowadays. The Mantel correlogram (Oden & Sokal 1986, Legendre and Legendre 1998; Borcard and Legendre 2012) is an obvious candidate method to estimate the relationship between synchrony and distance nonparametrically. The nonparametric covariance function (NCF) provide confidence intervals to be estimated by bootstrapping for a continuous function for synchrony as a function of distance (Bjørnstad et al. 1999b).

Spatial distance is the main focus in most studies of synchrony, with the expectation – and testing – that synchrony will decay with distance. However, distance is not the only variable to affect synchrony. In a heterogeneous landscape, there can be considerable dissimilarities in dynamics even between very close populations conditioned by different environmental factors. As a result, it is almost always the case that large portions of variation remain unexplained (Ranta et al. 1997a, 1998; Kendall et al. 2000; Hagen et al. 2008). Hence, it would be advantageous if the estimation procedure could account for variations caused by other important geographical variables that can also be obtained directly from maps. Altitude, for example, may also be expected to affect synchrony as altitude influences several climatic variables, such as temperature or rainfall (Hagen et al. 2008). Differences in altitude may potentially cause large variation in synchrony even among very close populations since climate effects such as rainfall can be dramatically different in lowlands and mountains (Pettorelli et al. 2005). Hence, the understanding of which sources of variation influence population synchrony is imperative in minimizing uncertainty. However, at present no such tool is available to ecologists.

The purpose of the present study is twofold. First, we analyse time series of abundances of the common vole *Microtus arvalis* (Pallas, 1778) to describe spatial population synchrony in the Czech Republic, central Europe. Second, by extending the non‐parametric covariation function, we develop a quantitative method that allows a dissection of the effects of additional variables such as altitude on synchrony. This method, the partial nonparametric correlation function (pNCF), is a generalization of the partial Mantel correlogram (Bjørnstad et al. 1995). We further explore how local indicators of spatial association (LISA) statistics can be extended to identify geographic areas of unusually high and low spatial synchrony. We performed this analysis because in Czech Republic we know that forests are common voles’ suboptimum habitats and consequently, their abundance in such habitats is expected to be reduced. Hence, we aim to test if forested regions are less synchronized than nonforested regions.

## Methods

We analysed spatiotemporal data for the common vole, *Microtus arvalis* in the Czech Republic, the most abundant vole in central Europe inhabiting open farmland and grassy habitats. As a proxy for population density in all 70 districts, we used abundance index: counts of active burrow entrances per hectare collected from 2000 to 2014. The entire data set consists of 70 districts although due to missing data only 49 districts were used in the analyses. The data were collected biannually for all districts with the spring data collected between March and April, and fall data between October and November.

The partial nonparametric covariance function is an extension of the partial Mantel correlogram previously proposed by Bjørnstad et al. (1995) to study evidence of genetic similarity as a function of habitat similarity while correcting for spatial distance, and follows Hall and Patil (1994) and Bjørnstad and Falck (2001) by estimating the underlying function of synchrony against distance using nonparametric regression. In the case of the partial covariance function the nonparametric regression is done on the residual relationship between synchrony and some second distance covariate. A bootstrap permutation test is used to test for significance due to the nonindependent nature of the three matrices (Legendre and Legendre 1998; Bjørnstad & Falck 2001).

We started by examining how Pearson's correlation between populations varies as a function of the geographical distance between them. We generated a matrix of cross‐correlation coefficients among the time series of abundance, and two distance matrices, one of geographic distances and one of altitudinal differences. We then applied a partial Mantel test to assess the relationship between altitude, geographical distances and correlation between populations. This test is appropriate to test the relationships among three distance matrices (Legendre and Legendre 1998). Subsequently we applied a modified nonparametric covariance function to produce direct estimates of the spatial covariance as a function of spatial distances, providing us with information on both local and regional synchrony. This function uses smoothing spline to regress the correlations on distances. To avoid the problem of nonindependence between time series and calculation of confidence intervals the analyses were bootstrapped using 500 iterations (Bjørnstad and Falck 2001; Bjørnstad et al. 2002; Liebhold et al. 2004). All computations were made with the package ncf which was modified to include a matrix of altitudinal differences. The modified correlogram, which we call the partial nonparametric covariance function (pNCF) calculates synchrony as a function of distance while correcting for some second covariate.

Subsequently, we used local indicator of spatial association (to estimate the local intensity of spatial synchrony. By decomposing global correlation into local neighborhood‐specific parts, LISA reveals local hot‐spots or cold spots. (Anselin 1995). LISA statistics give the spatially explicit strength of associations between neighboring districts allowing us to detect spatial clustering under the assumption that this is a nonrandom distribution of geospatial parameters (Anselin 1995). Since we are interested in patterns of synchrony among time series we use a multivariate extension which we call noncentered‐LISA (lisa.nc, ncf package, R Core Development Team 2005). We used 1000 resamples and a neighborhood size of 55 km which is close to the mean distance of 40 km between two neighboring district centers. This gave the mean number of five neighboring districts for each focal district which is very close to the observed mean number of neighboring districts. Additionally, in order to understand the pattern of local spatial correlations, the LISA results were regressed against district characteristics, such as altitude, climate variables proportion of forest cover. Forested habitats are suboptimum habitats and thus strongly avoided by common voles. We fitted a weighted linear regression model, with the number of data for the focal district as weights selected the best model according to the lowest AIC_c_, calculated in relation to an intercept‐only model.

## Results

**Figure 1:**
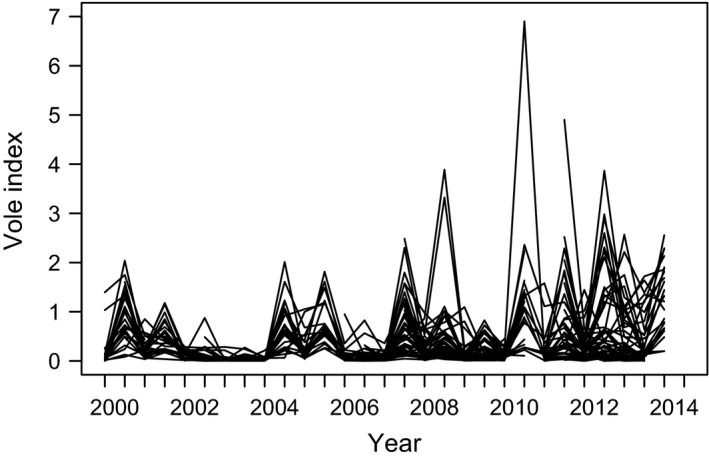
Population dynamics of the common vole for the 49 districts of the Czech Republic between 2000 and 2014. Vole index (in thousands) was measured as the number of active burrow entrances per hectare.

**Figure 2:**
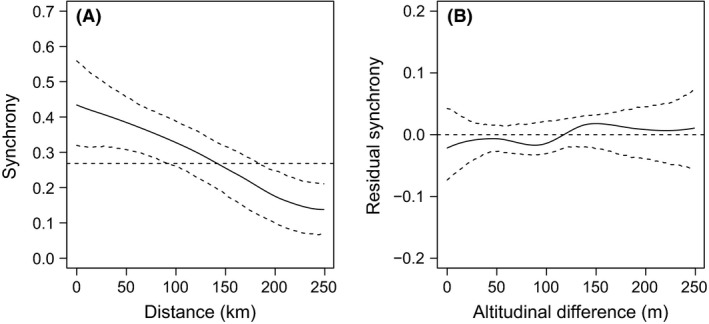
(A) The nonparametric covariance function of synchrony against distance estimated from vole abundances and (B) the partial nonparametric covariance function of synchrony against altitude when correcting for distance. The *y*‐axis in (A) shows the correlation. The *y*‐axis in (B) shows the residual correlation after accounting for the variation caused by distance. The upper and lower dashed lines in each plot represent the 95% bootstrap confidence intervals. The regional average synchrony is indicated by the horizontal solid line.

The common vole abundances fluctuated strongly (Fig. 1) and exhibited synchronous dynamics with the typical tendency for a decline of synchrony with increasing distance (partial Mantel correlation = 0.22, *P* = 0.002) but, not with altitude (partial Mantel correlation = −0.02, *P* = 0.2). As indicated by the nonparametric covariance function local synchrony reached the level of the average regional synchrony of 0.268 at a distance of approximately 150 km, but with no evidence of an altitudinal gradient (Fig. 2). We conclude that spatial variability in synchrony must be driven by some other landscape heterogeneities.

**Figure 3:**
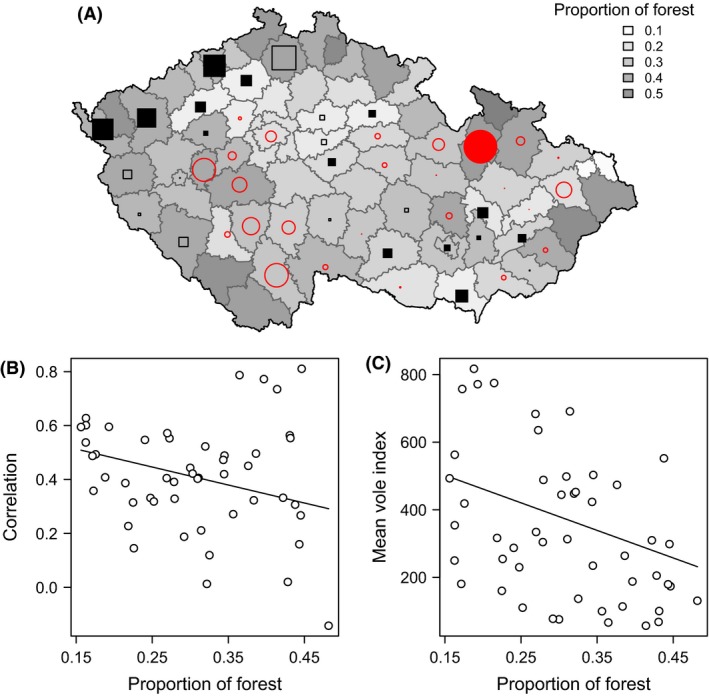
LISA, local indicator of spatial association superimposed on the map of the Czech Republic depicting the proportion of forested habitat (A). Negative (or below mean) values are signified by circles. Positive (or above mean) values are signified by squares. Values significant at a nominal (two‐sided) 5%‐level are represented by filled symbols, and non‐significant values by open symbols. The bottom panel show decreasing local district correlations from the LISA model with the proportion of forest (B) and decreasing mean population densities with increasing proportion of forest (C).

The quantification of spatial dependence using LISA indicated a significant clustering of spatially correlated districts in the western and south‐easterly region of the Czech Republic (Fig. 3). These correlations among neighboring districts can partly be explained by the degree of forestation. The higher the proportion of forested habitats in the district, the lower the degree of local synchrony (difference in AIC_c_ = 4.80; Fig. 3B) but not with district altitude (difference in AIC_c_ = 0.95). Typically, districts with more forested habitats are situated at higher elevations (correlation = 0.71, 95% CI 0.53–0.78) and inhabited by vole populations with lower densities (difference in AIC_c_ = 4.47; Fig. 3C). The effects of other explanatory variables, such as altitude, temperature, and rainfall variables, were not supported.

## Discussion

Spatial synchrony of population fluctuations, an ubiquitous phenomenon in nature, was shown to decrease with increased distance among populations. Despite the different methods used when studying synchrony, large proportions of variation still remain unexplained (Ranta et al. 1997a, 1998; Kendall et al. 2000; Hagen et al. 2008). In view of this problem, we extended the previously established non‐parametric spatial covariance function, to include further ecologically relevant geographic variables, such as altitude, a potential source of unknown variation. To illustrate the application of this method we used abundance data on the common vole from 49 districts and altitudes. We found that the common vole populations in the Czech Republic do exhibit strong synchronous dynamics with the typical tendency for a decline of synchrony with increasing geographic distance and weakened local synchrony in more forested areas. Despite the fact that we found no evidence of an altitudinal gradient in this particular study, perhaps due to the low range of altitudes among the districts studied, it is clear that this novel function can greatly increase our explanatory power when applied to populations distributed in a heterogeneous landscape with higher altitudinal rather than spatial scales.

We used the noncentered LISA statistics to study geographic variation in synchrony and show that forested regions are less synchronized than non‐forested regions. This is a multivariate extension of the LISA statistics commonly used in geo‐statistics (Anselin 1995), where correlations among time series are averaged within a spatial neighborhood. We used a randomization test to account for the nonindependence among the correlations.

Previous studies of synchrony in rodent dynamics have showed a high diversity of distance effects, from very strong distance decay among vole populations in Japan (Bjørnstad et al. 1999a) to very weak or no effect in Fennoscandia (Angerbjörn et al. 2001; Huitu et al. 2008). In the Czech Republic, we observed a moderate decay with geographical distance, where the regional level of synchrony was attained at a separation of around 150 km. Unlike distance, altitude did not affect vole population synchrony in the Czech Republic although it is often used as a proxy for climatic variables, such as temperature and rainfall (Hagen et al. 2008). Despite having some previous observational evidence that vole dynamics can be delayed toward mountains in the Czech Republic, the phase shift is perhaps not large enough to be proved. The absence of the altitude effect in this study might be explained by the fact that the range of about 350 m for altitudinal differences between the districts was too small. Perhaps much larger vertical heterogeneity is necessary for altitudinal decay in synchrony to be documented. Another potential cause of low sensitivity to altitude may stem from the large spatial scale at which the data were collected. The synchrony was measured among large areas, the districts, not among local populations. Irrespective of the obtained results, our hypothesis was under the assumption that along an altitudinal transect there are evident changes in the environment which may affect the distribution, occurrence and synchrony of the species under study.

The local indicator of spatial association showed higher degree of vole synchrony in the western and south‐easterly part of the Czech Republic. The correlation between the neighbor districts decreases as the proportion of forest increases. This observed trend is possibly due to the fact that forests are suboptimum habitats for the common voles. Highly forested districts thus typically harbor low‐density population suffering high sampling error (Lisická et al. 2007). Consequently, low‐density populations are prone to fluctuate erratically and exhibit less synchrony than high‐density ones. In fact, habitat heterogeneity and patchiness have long been considered as important influences on the occurrence and regularity of population cycles in voles (Spitz 1985; Stenseth 1985; Lidicker 1988). As observed by Delattre et al. (1992, 1996) the dominance of homogenous grassland in landscape structure favors common vole population outbreaks, whereas erratic dynamics can be expected in forested areas, perhaps as a result of reduced soil quality and plant productivity, consequent reduced reproduction and thus weaker responses to external environmental variation are followed by overall low population numbers. Our study indicates that the landscape composition, in this case the proportion of forest may be shaping the spatial distribution and consequently synchronizing dynamics of the common vole populations in the Czech Republic. Hence, the proportion of favored habitats at the regional scale should be considered when studying synchrony in cyclic microtine species (Delattre et al. 1996; Huitu et al. 2008).

The investigation of spatial patterns in animal populations is a key issue in ecology. Albeit the fact that the majority of studies are focused on testing hypotheses about which mechanisms are involved in shaping this patterns it is crucial to understand the sources of variation involved in the patterns of spatiotemporal dynamics in order to understand the underlying processes.

## Conflict of Interest

There is no conflict of interest among the authors.

## Supporting information


**Appendix S1.** R code.
