# Supplementary material for: Dissecting geographic variation in population synchrony using the common vole in central Europe as a test bed
Source: Ecol Evol. 2015 Dec 17;6(1):212–8. doi: 10.1002/ece3.1863 (PMC4716503; doi:10.1002/ece3.1863)
Supplement: Supplementary file 1 — Appendix S1. R code. [file ECE3-6-212-s001.docx]

**Appendix R code**

The partial nonparametric spatial covariance function builds on the nonparametric spatial covariance function of the NCF package.

Reading in data files on vole abundances and geographical coordinates

> data=read.table("data.txt", header=T)

> coord=read.table("coord.txt", header=T)

Calculating the 49x49 matrix of Pearson’s correlations among the 49 populations:

> cr=cor(data[,3:51], use="pairwise.complete")

Attaching the NCF package (for the gcdist-function)

> require(ncf)

We create a matrix for distances among populations. Since the coordinates are latitude and longitude we use greater-circle distance as implemented in the gcdist-function

> dis=matrix(0, ncol=49, nrow=49)

> for(i in 1:48){

+ for(j in (i+1):49){

+ dis[i,j]=dis[j,i]=gcdist(coord$lng[i], coord$lat[i], coord$lng[j], coord$lat[j])

+ }}

We have to make an ‘altitudinal distance’ matrix. We use absolute difference in altitude.

> alt=abs(outer(coord$a, coord$a, '-'))

The partial mantel test of synchrony against the other matrix (alt or dis) correcting for the third (dis or alt) is:

> partial.mantel.test(cr, alt, dis)

We use local polynomial regression to fit the nonparametric correlation functions This requires the locfit package:

> require(locfit)

For the regressions we need to make a data frame containing the upper triangle from each of the distance matrices and binding them in a data frame.

> sel=upper.tri(cr, diag=F)

> cdata=na.omit(data.frame(cr=cr[sel], dis=dis[sel], alt=alt[sel]))

We fit the spatial nonparametric correlation function

> lfitd = locfit(cr~lp(dis), data=cdata)

For the partial nonparametric correlation function we need the residuals around this synchrony versus distance relationship:

> resd=resid(lfitd)

> cdata$resd=resd

We can then look at partial nonparametric correlation of the residuals against ‘altitudinal dissimilarity’. Again we use local polynomials:

> ndat=data.frame(dis=seq(0,249, length=250), alt=seq(0,300, length=250))

> lfitresd = locfit(resd~lp(alt), data=cdata)

Pair-wise correlations are not statistically independent so we need to be careful in our resampling algorithm to construct bootstrap confidence intervals. We follow Bjornstad & Falck (2001) and use 500 resamples:

> bfitd=bfitresd=matrix(NA, ncol=250, nrow=500)

> for(i in 1:500){

+ res=sample(1:49, replace=T)

+ disB=dis[res, res]

+ crB=cr[res, res]

+ altB=alt[res, res]

+ cdataB=na.omit(data.frame(cr=crB[sel], dis=disB[sel], alt=altB[sel]))

+ cdataB=cdataB[cdataB$dis!=0,]

+ lfitdB = smooth.spline(y=cdataB$cr, x=cdataB$dis, df=sqrt(21))

+ resdB=resid(lfitdB)

+ cdataB$resd=resdB

+ lfitresdB = smooth.spline(y=cdataB$resd, x=cdataB$alt, df=sqrt(21))

+ bfitd[i,]=predict(lfitdB, x=ndat$dis)$y

+ bfitresd[i,]=predict(lfitresdB, x=ndat$alt)$y

+ }

We predict the observed nonparametric and partial covariance functions and erect 95% CIs for plotting:

> prd=predict(lfitd, newdata=ndat)

> presd=predict(lfitresd, newdata=ndat)

> cid=apply(bfitd, 2, quantile, probs=c(0.05, 0.95))

> ciresd=apply(bfitresd, 2, quantile, probs=c(0.05, 0.95))

> plot(ndat$dis, prd, type='l', xlab='Distance (km)', ylab='Synchrony')

> lines(ndat$dis, cid[1,],lty=2)

> lines(ndat$dis, cid[2,],lty=2)

> plot(ndat$dis, presd, type='l', xlab='Altitudinal difference (m)',

+ ylab='Residual synchrony')

> lines(ndat$dis, ciresd[1,],lty=2)

> lines(ndat$dis, ciresd[2,],lty=2)

To look for geographic hot-spots and cold-spots of spatial synchrony we use noncentered local indicators of spatial association as implemented in the lisa.nc()-function:

> require(ncf)

We first do some manipulation of the data

> zd=data[,3:51]

> na.fun<-function (x) length(na.omit(x))

> ndata=apply(zd,2,na.fun)

> z=matrix(t(zd), nrow=49,ncol=30)

> x=coord$x

> y=coord$y

We then carry out the lisa analysis. Significance is evaluated using a permutation tests with 10,000 iterations.

> mod2=lisa.nc(x, y, z, neigh=55000, na.rm = TRUE,

+ resamp=10000,latlon = FALSE, quiet = TRUE)

We can produce a bubble-plot of the lisa-statitics on spatial coordinates. The default plot shows below mean values are signified by red circles, and above mean values are signified by squares.

If a permutation test was performed, values significant at a nominal (two-sided) 5%-level will be represented by filled symbols and non-significant values by open symbols.

> plot(mod2)
